# Should I Stay or Go: Rural Ageing, a Time for Reflection

**DOI:** 10.3390/geriatrics3030049

**Published:** 2018-08-03

**Authors:** Emily M. Anderson, Sarah Larkins, Sarah Beaney, Robin A. Ray

**Affiliations:** College of Medicine & Dentistry, James Cook University, Townsville 4811, Australia; sarah.larkins@jcu.edu.au (S.L.); sarah.beaney@health.qld.gov.au (S.B.); robin.ray@jcu.edu.au (R.A.R.)

**Keywords:** ageing, rural, Australia, social support

## Abstract

(1) Background: Studies have shown that older people prefer to continue living in their own home and community as they age; however this is dependent upon available services and social support. In Australia about two thirds of people will age at home. The Australian Government provides home care packages to support ageing in place yet in rural areas not all services are available. The lack of employment opportunities in rural areas often results in family residing at a distance reducing available social support. This study aims to evaluate informal social support and its influence on ageing in place amongst older people in three Australian rural communities in Australia. (2) Methods: A multiple embedded case study was undertaken in three diverse rural communities. Eleven older rural residents ageing in place aged 65+ were interviewed about their ageing experience and plans for their future in the light of available social support along with 15 members of their social networks. Social networks were then visually depicted with the use of ecomaps and network members were interviewed. (3) Results show that kin and non-kin social networks support ageing in place however ageing is a time of change and reflection. (4) Conclusions: There is a need for more discussion within these networks when it comes to future planning.

## 1. Introduction

Ageing in place is defined as “the ability to live in one’s own home and community safely, regardless of age, income, or ability level” [1]. Numerous studies have shown that most people prefer to and plan to continue living in their own homes and community as they grow older [2,3,4]. In Australia the majority of older residents will age in place with one third requiring residential care [4].

The increasing age of the population in developed countries along with the increasing costs of residential care is causing governments to re-evaluate age care policy and implement reforms on long-term care. In Australia the increasing cost of residential care was outlined in a report produced by the Australian Research Council in 2014 that estimated that the costs of residential care comprised 70 percent of total aged care expenditure [5]. In response the Australian Government has been implementing changes to support ageing in place over the last 30 years starting with the introduction of the “The Residential Aged Care Reform Package” in 1997 which provided Community Aged Care Packages [6].

The most recent reforms outlined in the “Aged Care (Living Longer Living Better) Bill 2013” promise enhanced consumer choice for older people ageing in place structured over a 10 year period. This new bill offers consumer directed care but has also introduced asset tested co-payments along with the separation of the cost of care and accommodation whilst still capping the number of home care packages and residential care places [7].

Older people can access home care packages after an assessment of the required level of care has been made by the Aged Care Assessment Team. Four levels of care packages are available: basic care, low-level care, intermediate care and high-level care. Services consist of personal services, diet and nutrition, continence management, mobility, nursing and allied health services, transport and skin management. These are delivered by private service providers [8]. The number of home care packages is agreed yearly by the Government and they are allocated to each State/Territory.

A recent review of home care service provision showed that there is a current unmet demand for higher level packages with 67,000 older people on the wait list for intermediate and high level care. Of these 35,000 are currently in receipt of basic and low level care and are waiting on a higher level to become available [9]. Wait lists and the tightening of criteria for entry to residential aged care along with user pays charges have moved the responsibility for aged care support from government services to individual households with informal care provided by family and friends [10].

In line with the rest of the developed world, Australia’s population is ageing with adults over 65 estimated to comprise of 25% of the population by 2056 [11]. This age group will be over-represented in rural areas (possibly up to 36%) due to migration trends; with younger community members relocating to urban areas for work and education opportunities. Conversely many older migrants move to rural areas seeking a quiet retirement or for financial reasons due to housing affordability [12,13]. Much of the research on rural ageing uses a “marginalization” conceptual lens positioning older adults in rural communities as being at risk due to lack of access to health care and resources to meet their needs. Within Australia the term rural and remotes encapsulates all areas outside metropolitan areas, these areas are then further classified by distance from cities and local population size. This study uses the Modified Monash Model developed in 2015 which is used to map health care services along with health workforce models [14]. 

Small dispersed populations in non-metropolitan areas make the traditional healthcare workforce models difficult to implement and sustain leading to private service providers being reluctant to move into rural service provision due to higher running costs [15]. The lack of available local services results in more emergency room visits along with higher inpatient stays. Due to travel rural patients often receive inpatient for care for treatment that otherwise would have been provided as outpatient care [16,17,18]. Lack of aged care staff is a national concern with workforce shortages already evident in rural and remote areas due to lower wages and lack of training and development opportunities [19]. As younger families leave the area for work and education opportunities local services are further impacted due to decreased demand, this also decreases the available social support [13].

As the Australian Government moves towards a co-payment asset system this will disproportionally impact rural residents, as rurality is associated with lower incomes and higher rates of poverty with rural residents having lower educational qualifications and limited employment opportunities [20,21]. This lack of opportunity carries over into old age with lower superannuation and less savings to fund retirement [17]. There may also be a reluctance to claim government support due to stigma or lack of awareness of support available [22]. Many older rural women are more likely to live in poverty having never worked in paid employment, but spent years raising children, working on farms and supporting families. Life and work in Australia outside larger cities can be perceived as second rate, with the erosion of infrastructure and dwindling services experienced in rural areas [23].

One million Australians currently access age care services, however 80% of aged care support in the community is provided by partners, family, friends and neighbors [24,25]. Relationships are central to the ageing process and it is the friendships both kin and non-kin that support the ageing experience. The social relationships held within these networks can provide companionship, practical assistance and emotional support [15]. In gerontology social networks are considered as the mechanism through which support for ageing can be delivered as these networks contain the potential of support and care [26]. Each network holds the “assets” or social capital that has been amassed over the duration of the relationship built by trust, kinship and reciprocity. However the existence of a network tie does not guarantee support as the decision to provide support is multifactorial [27]. The social networks of older people are dynamic with losses and gains in the network throughout time: losses, such as death of a partner or siblings and gains such as consolidating new friendships [28].

Social networks have been studied extensively along with the beneficial effects of social connectedness in supporting healthy ageing [29,30,31,32]. Many studies on the ageing experience have looked at social networks showing an association between large social networks and improved health outcomes in later life [30,33,34]. However, the complex relationship between social connectedness and health (cognitive decline and overall mortality) has been inconsistent [35,36]. Nevertheless having an active social network and sufficient social support is linked with better self-rated health and increased wellbeing [37]. Given the perceived disadvantage experienced by older rural dwellers [2,38,39] deciding to age in place may not be the ideal option.

Litwak’s (1987) research on developmental lifespan proposed three main periods in older life when relocation is considered to support ageing. The first move is prompted by retirement and is influenced by lifestyle factors and choice. Retirees at this point are usually healthy and may decide to move to rural or coastal areas for the quieter lifestyle. The second move may occur due to loss of a partner or the need for support for health issues, at this point health may be compromised but can be supported by a mixture of informal and formal support. This move may be a move into town, retirement village or move to be closer to family. The third move is the move into formal care and may occur when the informal care network can no longer cope [40]. Carpenter in his study on the push factors of considering relocation in an ageing population showed that the ability to age in place and relocation decision is influenced by three main factors. Declining health, with physical impairments resulting in the loss of independence was the main reason for considering relocation. This was followed by financial concerns and lack of social support [41,42]. Restricted social networks can lead older people to consider relocation to move closer to family or to required support whereas the presence of larger integrated networks may support expectations of ageing in place [43].

Sociologists have studied the typology of social networks of older people to predict the support they may be able to access or determine well-being outcome measures [30,37,43,44]. These network typologies may indicate the sources, quantity and quality and type of available support. It is the available support in these networks that will impact on the decision to stay in the same place as opposed to relocate. The aim of this paper is to evaluate informal social support and its influence on ageing in place amongst older people ageing in place in three rural communities in Australia.

## 2. Methods

Case study design was chosen to capture the social phenomenon of ageing in place. The advantage of case design is that it allows for detailed participant narratives and documents both continuity and change [45]. Two sequential qualitative guided interviews were undertaken with participants with the second interview taking place approx. 14 months after the first interview. The findings from the first set of interviews are presented in this paper.

With no pre-determined hypothesis the case study design followed Stake’s (1978) conceptual framework. Using Stake’s approach a collective design of multiple embedded case studies was undertaken at three sites (each as a bounded unit) with the individual social networks including family and friends embedded within these [46,47,48]. All study sites were located in rural Queensland, and each site was characterized by a different industry, timber, sugar and cattle. The research team adopted a constructivist viewpoint in that that we as the researchers are endeavoring to interpret social reality by gathering knowledge and interpreting how ageing in place is constructed by our participants rather than discovered [49,50,51]. Throughout this study, we undertook a critical reflective approach to personal experience and expectations of ageing to minimize any bias.

### 2.1. Setting

Three sites in Table 1 were selected within rural Queensland two designated as category five and one category seven based on the Modified Monash Model (MMM) of rurality which takes into account remoteness and the size of the local population [52]. The MMM ranks areas from one to seven, with one being a major city and seven very remote. Remoteness classification is determined by the Australian Bureau of Statistics which uses the Australian Statistical Geography Standard-Remoteness Areas (ASGS-RA), residential population data from the 2011 Census to determine the five remoteness categories (RAs). The MMM uses the ASGS-RA data as a base, and further differentiates areas in Inner and Outer Regional Australia based on local town size. The category five sites were classified as both inner and outer regional rated five on the MMM, due to a location of more than 10 km road distance, of a town with population between 5000 and 15,000. The Category seven was classified as very remote and rated 7 on the MMM due to its geographical isolation. Sites were purposively selected due to remoteness and the diversity of each site, the researcher originally had four sites to include a mining town but was unsuccessful in recruiting. All sites were geographically distant from one another.

### 2.2. Recruitment

Ethical approval for the study was obtained from the James Cook University Ethics Committee H6262. Prior to this study the lead researcher met with an older local community member to discuss questions and issues included in the guided interview to ensure cultural appropriateness and relevance to older community. The research team then contacted local clubs, churches and doctor surgeries for approval to display information leaflets. Leaflets provided details about the study and the researcher’s contact details, interested participants were invited to contact the research team to arrange an interviews. 

Prior to each interview commencing participants were given the information sheet and after discussion of the project written consent was obtained. Inclusion criteria for primary network members were; aged 65 and over (there was no higher age exclusion) living in the community, with self-reported good health and sound cognitive function. Two validated screening tools were employed to check inclusion criteria, Lawton’s Instrumental Activities of Daily Living Scale (IADL) and General Practitioner Assessment of Cognition (GPCOG) [53,54]. Two participants were excluded during the screening process. Over the three sites 11 embedded case studies were recruited consisting of 26 members, after the inclusion of social network members, with 26 interviews being undertaken by the lead researcher alone (in some cases other family members were also present). After the primary interview and participants were asked if they were happy for the researcher to speak to a member of their social network, if consent was given the researcher then contacted the network member to see if they were happy to participate. If the participant was unsure information packs where then given to the participant to distribute to their social contacts with study and contact details requesting that they contact the researcher if they wish to participate. 

Network members interviewed consisted of partners, peers and younger family members.

Interviews were conducted at a venue of the participant’s choice, usually their home. Interviews were audio recorded with field notes and observations completed after each interview. Interviews covered four main areas linked to experience of ageing, reflections on ageing, support (received and given), home and social networks. The interview guide evolved during the study to reflect participant responses.

Eco maps were used as to provide a visual representation of the social relationships and the strength of these relationships [55,56]. Maps were assembled during the interview in a collaborative process with the primary participant starting with completion of member circles with categorization of relationship strength completed towards the conclusion when the interviewee was more comfortable by the use of lines [56]. These maps were then used to depict the social support available and allow classification into social support networks using Wenger’s typology [30] (see Table 2).

Data analysis occurred as an iterative process to inform subsequent interviews. Interviews were transcribed verbatim and interim summaries written up, with field notes and reflective remarks. Summaries were shared with the research team to identify any omissions or issues arising and facilitate discussion around each case [57]. Summaries and the original transcripts were then cross referenced when coding using NVivo 11 qualitative data analysis software (QSR International, Melbourne, Australia) to ensure that all key issues and researcher’s reflections were captured. 

Data analysis was based on early steps of grounded theory methodology. A first round of open coding was undertaken with new nodes being added as more interviews were undertaken, as the analysis progressed these were then coded into individual categories (concepts). Emerging concepts were then discussed with the research team and where required audio recordings reviewed to ensure validation, this was important as although interviews were transcribed verbatim with some comments “tone” was important to capture. Themes were analyzed both within cases and across cases [57]. To ensure authenticity, initial interviews were independently coded by two team members, themes discussed with the research team and validated across interviews.

## 3. Results

**Figure 1:**
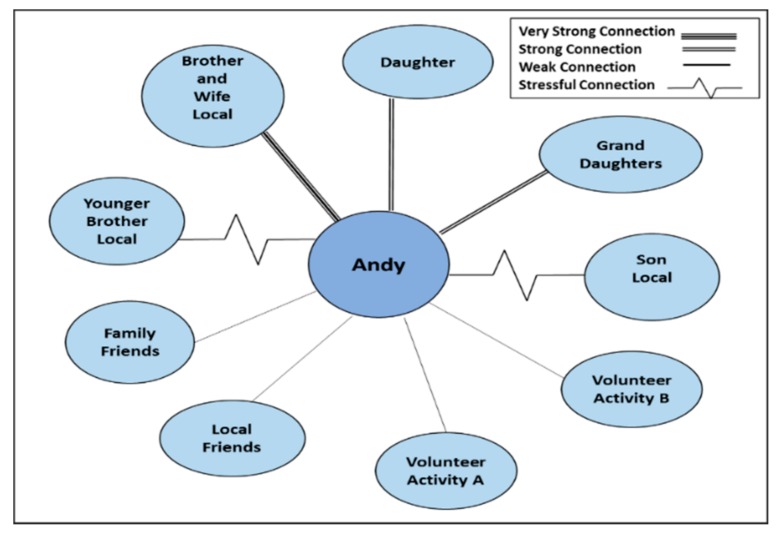
Restricted Family Dependent Network, Andy Milltown.

A total of 11 older participants (4 male, 7 female) were interviewed over the three sites aged 71–92 years. In addition 15 social network members were also interviewed which included partners, family and friends aged between 26 and 80 years. Aliases are used for confidentiality. Firstly network structured will be reported followed by factors that impact upon relocation. Figure 1 below shows Andy’s restricted family dependent network.

### 3.1. Social Networks

Network types were independent of location with the majority being integrated networks. Within these networks wider community focused networks were more common due to family residing at a distance. In Cattletown, adult children and siblings still lived rurally, yet the distance was still a limiting factor due to large dispersed cattle properties.

If anything happens to one or both of us the children would have to get on a plane to come up and see what are those two up to now, you know, what have they done this time. Whereas if we’re in City A or City B they’ve only got to hop in a car.

(Danny, 70, Milltown, married, Eddie’s friend)

Interestingly, in community focused networks some networks hubs had a closer connection to non-kin members, the presence of a family member within this network did not immediately correlate to the strongest connection. Studies on kin and no-kin networks have shown a more beneficial effect of networks with non-kin resulting in decreased mortality and nursing home admissions for those with a close confidante [58]. Close confidantes played an important role in social networks with one participant, although part of a large integrated network, confessing her unhappiness with a lack of a close confidante. The importance of close friendships was commented on in all integrated networks, this was especially important in dealing with the loss of a partner.

She was there for me when my husband died, I was there for her when hers died.

(Tessa, 65, Milltown, Single, Sophie’s friend)

The three restricted networks (one private restricted and two family dependent) had males at their centre. Although women tend to have larger social networks than men research has shown that even after adjusting for social factors it is unlikely that these networks are a result of gender alone [34]. All three of the primary network members self-identified as being a loner and preferring their own company from a younger age. Two were currently living with a female partner with most social activities and interactions taking place in the home environment and the other had lost his partner the year before.

Cloutier-Fisher’s work on small social networks discussed the importance of situating the social network in the context of the individual’s life course with small social networks not an indication of loneliness, but a function of their personality rather than due to gender or increasing age [59].

Mark related an incident at boarding school that he felt was the start of him withdrawing and becoming a loner. He volunteers occasionally at the local school and undertakes self-directed community services at his own pace and under his “own terms”.

I’m giving back a little but only when they ask for it.

I haven’t got family here, I haven’t got too many friends and would rather organise ad hoc paid care than be pushed by bureaucrats (Mark discussing whether he would use government home care services).

(Mark, 75, Milltown, Partnered, Primary)

Reg lives at home with his wife and his four children all live close by with their children. The family is very close and supportive of one another and mainly socialise together and states “he is allergic to clubs” and likes to do his own thing.

Reg can be a bit anti-social—I don’t want to go there—but if we go to a party he is the last one to leave.

(Marnie, 57, Sugartown, Reg’s wife)

In the remaining family dependent network Andy had been widowed the previous year, close network members consisted of a daughter residing at a distance and one close kin member in the rural town who was caring for their own partner. Andy volunteered at the local library an activity he had become involved in through his wife.

Your wife’s doing something and you just get drawn into it.

(Andy, 76, Milltown, widowed, Primary)

Whilst these relationships provided emotional support there was not the capacity for provision of instrumental support and was unsure how he would cope if his health deteriorated.

Members of these networks enjoyed low levels of interactions with the community and volunteered under their own terms.

### 3.2. Stay or Go

*…“we’ll have to think about what the next stage is, unless we die first, which happens to all of us isn’t it. You’ve got to plan unless you die first.”*

(Danny, 70, Milltown, Eddie’s Friend)

Within this study there were two types of relocation; proactive and reactive moves. A previous study has shown that the main push factors for considering relocation in older communities was increasing health needs (65%) followed by finances (26%) and social isolation (6%) [41]. Of the 11 embedded cases two (Reg and Clare) were actively relocating, five were considering a future move, one had relocated in the last 6 months and three had no current plans for relocation.

Reactive relocation occurred in two participants associated with a decline in health forcing a move to a more supportive location, due to both living both rurally and remotely. One participant stayed within the local community and another moved to the regional city. Reg relocated to the regional city due to complex health needs and to be closer to family. He expressed regret in leaving the move too late and now being unable to cope with managing the rural property (which now had to be sold) and although resigned to the move was not happy in the city.

…if we get around to listing it, it is a bit of a mess it has overgrown and is going to have to be sold overgrown,—I always said I was worried we would leave it too late now we left it too late, not capable of doing it used to be able to.

15 acres compared to this, there is no comparison this is like going to hell without dying (Reg on now living in the city).

(Reg, 72, Sugartown, Married, Primary)

His daughter expressed family concern with the previous location and relief that they were nearer to specialized health services and that all children could now provide more support to both parents.

…rural hospital which they do their best they can but don’t have the facilities of city hospital us kids couldn’t lend a hand to look after Dad if he was sick if Mum was busy working.

(Susie, 31, Sugartown, married—Reg’s daughter)

Beth had moved within the last six months from her rural property. This move was reactive brought about by ill health and family concerns with regards to isolation of her rural property and how she would cope. Her move was organized by family and friends who packed up the house and arranged a rental property in the rural town.

I was going through a bit of a low health period at the time and they decided I wasn’t fit to live on my own and anyway, [supportive friend] had offered this house to me before, so I moved in to town.

(Beth, 94, Cattletown, widowed, primary)

The most common relocation discussed in the first part of this study concerned a proactive move in order to keep independence for longer. When deciding possible relocation from their current home participants reflected on the difficulties of home upkeep, expectations of family care, community and availability of formal services. Participants planning to move expressed the difficulties of selling in rural areas, with properties taking a long time to sell and the difference in property values with cities having higher property values.

Our son and his family lives in regional city that pull us to there but there are lots of problems getting there, two of which are house sales in small towns isn’t too active and then the price that you get for your house compared to the price that you have to pay for some accommodation in regional city.

(Cate, 75, Sugartown, married, Libby’s friend)

We’ve given it a sort of two year timeframe to sell and we would probably try and buy something or build in walking distance of town.

(Clare, 74, Milltown, married, primary)

### 3.3. Home Upkeep

Although the study is based around three towns many of the participants lived on the outskirts or satellite settlements. All participants lived on larger rural blocks or detached homes and for some this was seen as a possible limitation for the future whilst others were already experiencing problems.

…so we got this block of land which has got a lot of forest around it and we’re just sort of coasting along really until we get probably close to 80 when we physically won’t be able to handle this place anymore and we’ll have to think about what the next stage is.

(Danny, 70, Milltown, married, Eddie’s friend)

### 3.4. Families

Relocation was framed around future health and support needs and where would be the best place to access them. When considering a future where care may be required an expectation of some form of family support was the norm with most cases discussing relocation from the community moving nearer to family:

My son thinks I should go to his town, my daughter thinks I should go to her town. While I’m as fit as I am I’ll stay here but if I deteriorate, if I needed full time care, I would definitely go to his town or her town.

(Beth, 94, Cattletown, widowed, primary)

It would be easier for my daughter when I needed her to do things for me and that. She’s working so I can’t drag her away from her job.

(Jess, 74, Milltown, Mark’s partner)

Support expected by family varied in each case as did acceptance of types of support financial aid and personal care. Many participants expressed the desire not to “become a burden” and that although they expected care that care was to be emotionally supportive to “look out for them” rather than look after any physical needs.

My family gives me great support, yes, but I wouldn’t live with them. We’ve got an aged care centre here, I would go there if I couldn’t look after myself.

(Caitlin, 82, Cattletown, widowed, primary)

…when he has been really sick he has been ok with people helping him but he is pretty quick to get back on his feet he is very proud he doesn’t want someone to do something he can do himself but when he can’t do something he will let someone help him.

(Susie, 31, SugarTown, Reg’s daughter)

### 3.5. Home Care Services

All participants reported knowledge of existing at-home aged care services and felt that those services would support them to remain in the community. The knowledge of and provision of home aged care packages was positive and at home services were used in all three sites.

I’d like to stay in my own home and I know that we can get support like that.

(Eddie, 74, Milltown, widowed, discussing at-home services)

That lady that I just spoke to on the phone, she just can’t get over how wonderful HACC is here, how accommodating they are.

(Carrie, 71, Cattletown, widowed, primary)

If I had to have someone come in to do a bit of housework or something like that I’d accept it, yeah. Because I know as I say with Mum, they were just so good and they become part of the family. (Jenny on her previous interaction with at-home services, when they looked after her mother).

(Jenny, 72, Milltown, Married, Eddie’s friend)

Relocation to residential aged care was discussed but was not judged desirable by the participants with one participant describing it as “final punishment for being old”. Others were more pragmatic describing residential homes as being necessary ‘for people who didn’t have anywhere else to go” and “it is a thing you need to have in mind”. In all but one case residential care was seen as last resort. Jorgensen (2009) demonstrated that older adults had little decisional control over relocation to residential care with the decision being made by their families and professionals [60]. This was alluded to in the way participants spoke about entry into residential care as being a forced move, taken out of their hands rather than a chosen move, Debbie discussed leaving the decision on this to both her children acting on her behalf.

I think they’re great for people who want to downsize and all that sort of stuff, but for me, only if it was forced.

(Debbie, 85, Sugartown, widowed, primary)

My sister and I talk about getting old and we hope we don’t have to go to the old age home. Like maybe have a quick big heart attack and gone.

(Jess, 74, Milltown, Mark’s partner)

…like Mum, she never wanted to be—and her pain, she was so sick in the end that they wanted to put her into care and thank goodness, thank the Lord she passed away.

(Jenny talking about her mother’s illness, Milltown, 72, married, Eddie’s friend)

Only Libby with comprised health felt that residential would be suitable for her but not did not wish to leave her husband Dave who wanted to age at home. Their current home is a small beachside settlement about twenty kilometers from the nearest rural town, they had previously tried to sell their home but had been unsuccessful.

My husband you know he wants to stay here so…uh if I had a choice or the doctor said to me you would be better off in a home well inside I would be laughing when do we go?

So I think that the government want you to stay in your home for as long as possible and there is nowhere to go, there is a waiting list, virtually someone has got to die.

(Libby, 77, Sugartown, married, primary)

In a few cases there were differing views within the network with regards to the location of ageing with partners and children having different views and planning on ageing in different locations. In one case the children had suggested a relocation plan without any discussion on the desires of the parent which had caused conflict in the relationship.

So I don’t think it’s in the plan of things that my family will be—they’re there but they’re certainly not going to be looking after me.

(Carrie, 71, Cattletown, widowed, primary)

Mismatch in expectations of relocation between a daughter and parent was shown in another case where the parent expressed a desire to move into a granny flat and the daughter would prefer assisted living accommodation. Couples also named different locations as to personal preference to age with one partner naming one family member to relocate to and the other naming another. Both couples had partners on reflection they may have answered the question thinking about the loss of their partner and what they would do then.

They’re solid so he’d probably live there if anything happened to me but I wouldn’t, I’d live here.

(Clare, 74, Milltown, married, primary)

For some the rural aspect meant that they were reliant on being able to drive to stay in their current home—public transport was limited in all locations and for those participants living on large rural blocks non-existent. For these participants loss of their driving license was related to loss of their independence and access to local services. For Clare and her husband this prompted the move to the rural town to continue to access services and keep connected with her social network.

I mean you can lose your licence and you can’t go anywhere and he said he doesn’t want to be dependent on other people. It’s not what we want to do, it’s just being sensible.

(Clare, 74, Milltown, married primary)

For Sophie, although living in town, a lack of transport was a factor in considering future relocation to maintain independence.

I’d just like a bus, I’d just like to be able to get out of this place without relying on somebody else … to say I’ll get the bus.

(Sophie, 73, Milltown, married, primary)

Throughout this interviews reciprocity in driving was a theme between friends and families whilst the recipients were in need of these services there was a theme that they didn’t like to ask or be “a burden”.

## 4. Discussion

Much of the research into social networks has been on care required and older people with chronic conditions. In contrast, this study has involved healthy community residing individuals who form the majority of older people in Australia [27]. This study shows that older people are constantly reflecting on their environment and whether it is the right fit for them and their needs. It looks at the impact of social networks and local community services when considering whether to age in place. Assessment of local support that they can access and how others have managed is essential for future planning. This is in contrast to the widely held view that ageing is a time to reflect on the past and older people have to plan for the future, which may include relocation to allow them to maintain their independence [61]. 

Although social networks do not guarantee the provision of support, they are the vehicle that holds the potential of informal support for ageing in place and this potential capital needs to be accounted for when considering a relocation [27]. Results show that friends provide a lot of emotional support especially in the absence of family due to living at a distance, although strong family connections were still shown. Peer friendships especially including a close confident have been shown to have a protective effect on mortality [58,62]. However friends and confidantes in this study tended to be of a similar age which may limit the potential of available instrumental support [40]. 

Social networks also increased the awareness of available formal support that may influence the decision on whether or not they would be able to stay in their own homes. Knowledge of availability of home care services is a major factor when making the decision whether to stay in the community [43]. Most participants in this study were in their late seventies and were aware of the formal local aged care support services from their peers and discussed how these services might be relevant to help them age in place. Some participants had already accessed forms of formal support to implement modifications to their home. Monitoring how their peers coped with losses or shifts in independence also helped participants decide on what would or indeed would not be suitable for them [41].

Within this study there was an expectation that in the event of age related illness or decline that family, mainly adult children would be on hand to provide support. This is the normative expectation with adult children continuing to be the main providers of age related health and social support to their parents with more than half of middle aged daughters called on to provide care for their ageing parents [63]. However accessing this support was not taken lightly with many naming it as a last resort and putting limitations of the type of support they would accept from their children. No personal or financial support from children would be accepted with the overall theme of “not wanting to be a burden”. Whilst it was expected that the children provide support there was no clear concept of what that support would be. There were comments on how busy their children and grandchildren’s lives were, distance as a barrier, and the fast pace that life was lived and it may be that this “generational observing” lowered their expectations of available care [64]. Whilst the majority of adult children accept there is an obligation for them to provide support for their parents there was no agreement on what this support should consist off [65]. 

Much of the research into rural ageing has been concerned with inferior service and delivery of health care using a marginalization lens however to healthy community residing adults this does not impact upon their views on the suitability of their community [66]. This was consistent with the findings of this study. Suitability of the community may change in response to health declines and the need for increased use of local health services. This is the uncertainty of ageing in that older adults most constantly reflect on what the future may hold and take action to maintain their independence. We can never know the future. It should be noted that not all older people have the resources to maintain their independence, finances to move, a social network or family to support them. Some participants in this study had no contingency plans if they were unable to manage in their own home and expressed a desire to die in their own home. For this group the future holds uncertainty and a dramatic increase in care requirements may force a crisis point leading to entry into long term residential care.

The limitations of this study are that it is a small qualitative study undertaken in three rural communities in Australia which may limit transferability of these findings. However although each community was diverse in both economy and geography, participants described similar experiences and challenges. In addition resonance with the literature and studies in other developed countries might suggest that the findings are applicable elsewhere. 

## 5. Conclusions

Rural communities offer social support with many older people being part of integrated social networks. Older people with no health problems are well supported in these communities. Whilst there is an expectation on the family to provide support to allow ageing in place there is no agreement on what form this support should take. When considering relocation proactive moves are made with the purpose of maintaining independence, lifestyle or moving closer to family. Reactive moves occur in response to a decline in health when there is a need to relocate to health based services and available social network support. This study shows the need to promote and facilitate conversations of care expectations of social networks at community level to allow for proactive ageing in place.

## Figures and Tables

**Table 1:** Study Sites.

Town	Population	Geography	Remoteness Area	MMM
Sugar Town	4767	Coastal	RA 2-3	Category 5
Cattle Town	1152	Outback	RA 5	Category 7
Mill Town	1442	Rain Forest	RA 2-3	Category 5

**Table 2:** Network Typologies.

Integrated Social Networks	Characteristics	Embedded Cases
Locally Integrated	Typically large social networks characterized by close connections with family, friends, neighbors and the community.	Debbie, Caitlin, Sophie
Wider Community Focused	Lack of local family members but contact with friends and neighbors. Have extensive contact with relatives who live some distance away (adult children, siblings)	Carrie, Clare, Beth, Eddie, Libby
**Restricted Social Networks**		
Private Restricted	No local family, few friends and few links to community (may be married).	Mark
Family Dependent	Networks contain mostly family with only a few friends and neighbors.	Reg, Andy
Local self-contained	Solitary, little contact with family or community, may receive some help from neighbors.	

Source: [30].
